# *Pf*MDR2 and *Pf*MDR5 are dispensable for *Plasmodium falciparum* asexual parasite multiplication but change *in vitro* susceptibility to anti-malarial drugs

**DOI:** 10.1186/s12936-015-0581-y

**Published:** 2015-02-14

**Authors:** Maarten van der Velden, Sanna R Rijpma, Frans GM Russel, Robert W Sauerwein, Jan B Koenderink

**Affiliations:** Department of Pharmacology and Toxicology, Radboud University Medical Center, Nijmegen, The Netherlands; Department of Medical Microbiology, Radboud University Medical Center, Nijmegen, The Netherlands

**Keywords:** ABC transporter, MDR, *Pf*MDR2, *Pf*MDR5, Anti-malarial, *Plasmodium falciparum*, Malaria

## Abstract

**Background:**

Membrane-associated ATP binding cassette (ABC) transport proteins hydrolyze ATP in order to translocate a broad spectrum of substrates, from single ions to macromolecules across membranes. In humans, members from this transport family have been linked to drug resistance phenotypes, e.g., tumour resistance by enhanced export of chemotherapeutic agents from cancer cells due to gene amplifications or polymorphisms in multidrug resistance (MDR) protein 1. Similar mechanisms have linked the *Plasmodium falciparum Pf*MDR1 transporter to anti-malarial drug resistance acquisition. In this study, the possible involvement of two related MDR proteins, *Pf*MDR2 and *Pf*MDR5, to emerging drug resistance is investigated by a reverse genetics approach.

**Methods:**

A homologous double crossover strategy was used to generate *P. falciparum* parasites lacking the *Pfmdr2* (*Pf*Δ*mdr2*) or *Pfmdr5* (*Pf*Δ*mdr5*) gene. *Plasmodium* lactate dehydrogenase activity was used as read-out for sensitivity to artemisinin (ART), atovaquone (ATO), dihydroartemisinin (DHA), chloroquine (CQ), lumefantrine (LUM), mefloquine (MQ), and quinine (QN). Differences in half maximal inhibitory concentration (IC_50_) values between wild type and each mutant line were determined using a paired *t*-test.

**Results:**

Both *Pf*Δ*mdr2* and *Pf*Δ*mdr5* clones were capable of asexual multiplication. Upon drug exposure, *Pf*Δ*mdr2* showed a marginally decreased sensitivity to ATO (IC_50_ of 1.2 nM to 1.8 nM), MQ (124 nM to 185 nM) and QN (40 nM to 70 nM), as compared to wild type (NF54) parasites. On the other hand, *Pf*Δ*mdr5* showed slightly increased sensitivity to ART (IC_50_ of 26 nM to 19 nM).

**Conclusion:**

Both *Pfmdr2* and *Pfmdr5* are dispensable for blood stage development while the deletion lines show altered sensitivity profiles to commonly used anti-malarial drugs. The findings show for the first time that next to *Pf*MDR2, the *Pf*MDR5 transport protein could play a role in emerging drug resistance.

**Electronic supplementary material:**

The online version of this article (doi:10.1186/s12936-015-0581-y) contains supplementary material, which is available to authorized users.

## Background

ATP binding cassette (ABC) transporters are membrane-bound proteins that translocate a wide variety of substrates, including sugars, peptides, inorganic ions, proteins, and drugs across membranes by consumption of ATP, even against high concentration gradients [1,2]. These transport proteins either consist of two domains, a transmembrane domain with six membrane-spanning α-helices and a nucleotide-binding domain (half transporter) or four domains, two from each of the above alternating each other (full transporter). The nucleotide-binding domain is characteristic for ABC transport proteins and contains conserved Walker A and B motifs to bind ATP and an ABC signature sequence [3,4]. It is well described that the human P-glycoprotein, a member of the B subfamily of ABC transport proteins, also known as multidrug resistance (MDR) proteins, provides tumour resistance to chemotherapeutics by extrusion of these substances from cancer cells due to *MDR1* gene amplifications or polymorphisms [2,5-7]. Based on sequence identity, *Plasmodium falciparum* has been shown to contain 16 ABC family members in its genome [8], some of which also play important roles in drug resistance development in this parasite.

*Plasmodium falciparum* causes the most dangerous type of malaria, a life-threatening disease that affected 198 million individuals with an estimated 584,000 fatalities in 2013, especially African children under five [9]. Although treatable, recent reports showed emerging resistance as measured by prolonged parasite clearance times against artemisinin, the fast-acting drug in the first-line artemisinin-based combination therapy (ACT), on the Thai-Cambodian border and in Western Cambodia [10,11]. Whereas mutations in the K13-propeller have been identified as molecular markers for increased parasite half-life, additional loci involved in artemisinin resistance are yet to be discovered [12]. Furthermore, amplification of and polymorphisms in the *P. falciparum mdr1* and *mrp1* genes have been associated with, e.g., artemisinin, chloroquine, lumefantrine, mefloquine, and quinine resistance [13-18]. In addition, polymorphisms and differential Asn-repeat lengths found in *Pfmdr6* were shown to alter drug sensitivity to artesunate and piperaquine [19-22], while a single mutation in the heavy metal transporting *Pf*MDR2 [23] was linked to *in vitro* pyrimethamine resistance, which however, depended on *dhfr* mutation status [24]. These findings show an important role for ABC transport proteins in *P. falciparum* drug resistance acquisition. Although the contribution of the MDR family in emerging drug resistance seems evident, as of yet no studies have assessed the effect of *Pfmdr* deletion lines on altered drug sensitivity.

Here, using a reverse genetics approach, both *Pfmdr2* and *Pfmdr5* were targeted for gene deletion. Subsequently, the involvement of two *P. falciparum* genes, *Pfmdr2* and *Pfmdr5,* on drug sensitivity to seven well-established anti-malarial drugs, including artemisinin (ART), atovaquone (ATO), dihydroartemisinin (DHA), chloroquine (CQ), lumefantrine (LUM), mefloquine (MQ), and quinine (QN), belonging to four different drug classes (4-aminoquinolines, artemisinins, aryl-amino alcohols, and naphtoquinones), was studied [25].

## Methods

### Parasite cultivation

*Plasmodium falciparum* wild type (NF54) and mutant lines *Pf*Δ*mdr2* and *Pf*Δ*mdr5* were cultured in a semi-automated system as described previously [26,27]. Briefly, parasites were grown *in vitro* in 5% haematocrit in RPMI medium supplemented with human serum (complete medium), which was changed twice daily. Human red blood cells were refreshed weekly and obtained from the Dutch national blood bank (Sanquin, Nijmegen).

### Generation and genotyping of *Pf*Δ*mdr2* and *Pf*Δ*mdr5* parasites

*Plasmodium falciparum mdr2* and *mdr5* genes were stably deleted according to a homologous double crossover strategy [28,29]. Deletion constructs were based on the reported pHHT-FRT-(GFP)-*Pf52* construct in which the homologous regions were replaced with *mdr2* and *mdr5* target regions (TRs), respectively [30]. These regions were generated by amplifying *P. falciparum* NF54 genomic DNA (gDNA) using *PfuUltra* II Fusion HS DNA Polymerase (Bio-Connect B.V., Huissen, The Netherlands) with primers (P) P9 and P10 for the 5′ target region and primers P11 and P12 for the 3′ TR of *mdr2*, respectively. Similarly, primers P13-P16 were used to generate TRs for *mdr5* (Additional file 1: Table S1). Following TOPO TA subcloning (Life Technologies Europe B.V., Bleiswijk, The Netherlands) of the amplified TRs, these were validated by restriction digestion and sequencing. Then, 5′ and 3′ TRs from each *mdr* gene were cloned in the pHHT-FRT-(GFP)-*Pf52* construct using *BssHII* plus *BsiWI* and *XmaI* plus *NheI* restriction enzymes, respectively. This resulted in two deletion constructs, pHHT-FRT-(GFP)-*Pfmdr2* and pHHT-FRT-(GFP)-*Pfmdr5*. Transfection and selection were performed as previously described [30], resulting in two *mdr* mutant lines. Both lines were subsequently transfected with the pMV-FLPe construct in order to excise heterologous DNA (h*dhfr::gfp* selectable marker) flanked by flippase recognition target (FRT) sites using enhanced flippase (FLPe) recombinase [30]. Following limiting dilution, two cloned mutant lines, *Pf*Δ*mdr2* and *Pf*Δ*mdr5* were derived for downstream analysis.

Genotype analysis of the parasite mutants was performed using Expand Long Range dNTPack (Roche Diagnostics Nederland B.V., Almere, The Netherlands) (LR-PCR). Mixed asexual blood stage gDNA from wild type and deletion parasites was isolated using the QIAamp DNA Blood Mini Kit (Qiagen N.V., Venlo, The Netherlands). Primers P1 and P2 plus P5 and P6 (Additional file 1: Table S1) designed to flank the 5′ and 3′ TRs for *mdr2* and *mdr5*, respectively, were used to analyse wild type and mutant DNA using LR-PCR for correct double homologous crossover integration. The LR-PCR started with an initial denaturation at 94°C for 5 min, followed by 35 cycles of denaturation at 94°C for 30 sec, annealing at 43.5-50°C (43.5°C for wild type and mutant *mdr2*, 46°C for wild type *mdr5* and 50°C for mutant *mdr5*) for 30 sec, elongation at 60°C for 15 min [31] and a final elongation step at 62°C for 15 min. An additional intra-exonic PCR was performed on gDNA using primers P3 and P4 and P7 and P8 designed within the gene exon of both *mdr* genes (Additional file 1: Table S1). Briefly, gDNA from wild type and mutant lines was isolated as described above and amplified using *Taq* DNA polymerase (Life Technologies Europe B.V., Bleiswijk, The Netherlands). Denaturation at 94°C for 2 min was followed by 40 cycles of denaturation at 94°C for 15 sec, annealing at 52°C for 45 sec and elongation at 60°C for 30 sec, followed by a final elongation step at 60°C for 2 min.

### Anti-malarial sensitivity assays

ART, ATO, CQ, DHA, LUM, MQ, and QN were all purchased from Sigma-Aldrich Chemie B.V. (Zwijndrecht, The Netherlands). ART and QN were dissolved in methanol, ATO, DHA, LUM, and MQ were dissolved in DMSO, CQ was dissolved in complete medium (described above) and serial dilutions of all drugs were stored at −20°C and thawed prior to use. Anti-malarial sensitivity assays were performed in at least three independent consecutive experiments using a slightly modified *Plasmodium* lactate dehydrogenase (pLDH) method as described previously [32]. In short, 50 μL of serially diluted drugs was added to black, clear-bottom, 96-well, cell culture plates (Greiner Bio-One B.V., Alphen a/d Rijn, The Netherlands) in triplicate (duplicate for the 0 concentrations). Then, 50 μL mixed asexual stage wild type (NF54) and mutant parasites (2.5% parasitaemia in 1% final haematocrit) was added to each drug-containing well. Following incubation for 72 hrs at 37°C in a candle jar [33], the 96-well plates were frozen for at least 3 hrs at −20°C. After thawing the plates, parasite pLDH activity was measured by adding 70 μL of freshly made reaction mix (286 mM sodium L-lactate (Sigma-Aldrich Chemie B.V., Zwijndrecht, The Netherlands), 286 mM 3-acetyl pyridine adenine dinucleotide (Sigma-Aldrich Chemie B.V., Zwijndrecht, The Netherlands), 357.5 μM resazurin (Sigma-Aldrich Chemie B.V., Zwijndrecht, The Netherlands), 5.66 U/mL diaphorase (Worthington Biochemical Corp., Lakewood, NJ, USA), 1.4% Tween-20 (Sigma-Aldrich Chemie B.V., Zwijndrecht, The Netherlands), 20 mM Tris–HCl pH 8.0) to each well. Next, the plates were incubated in darkness for 30–60 min and absorbance was measured at 590 nm after excitation at 530 nm using a Synergy 2 Multi-Mode Microplate Reader (Bio-Tek, Bad Friedrichshall, Germany). The logarithm of half maximal inhibitory concentration (logIC_50_) values were determined by non-linear regression fitting of dose–response inhibition curves with variable slopes using GraphPad Prism version 5.03 (GraphPad Software, Inc., La Jolla, CA, USA). For each anti-malarial drug, Hill slopes were fixed to the average of all non-ambiguous slopes for each *mdr* mutant and their wild type control. Comparisons for significant differences between the obtained logIC_50_-values of wild type and mutant groups were carried out by a paired *t*-test. Finally, the plotted curves represent the combined averages of each individual anti-malarial assay per line, where the top of each curve fit was set to 100%.

### Results

#### *Plasmodium falciparum mdr2* and *mdr5* are dispensable for asexual blood stage multiplication

**Figure 1:**
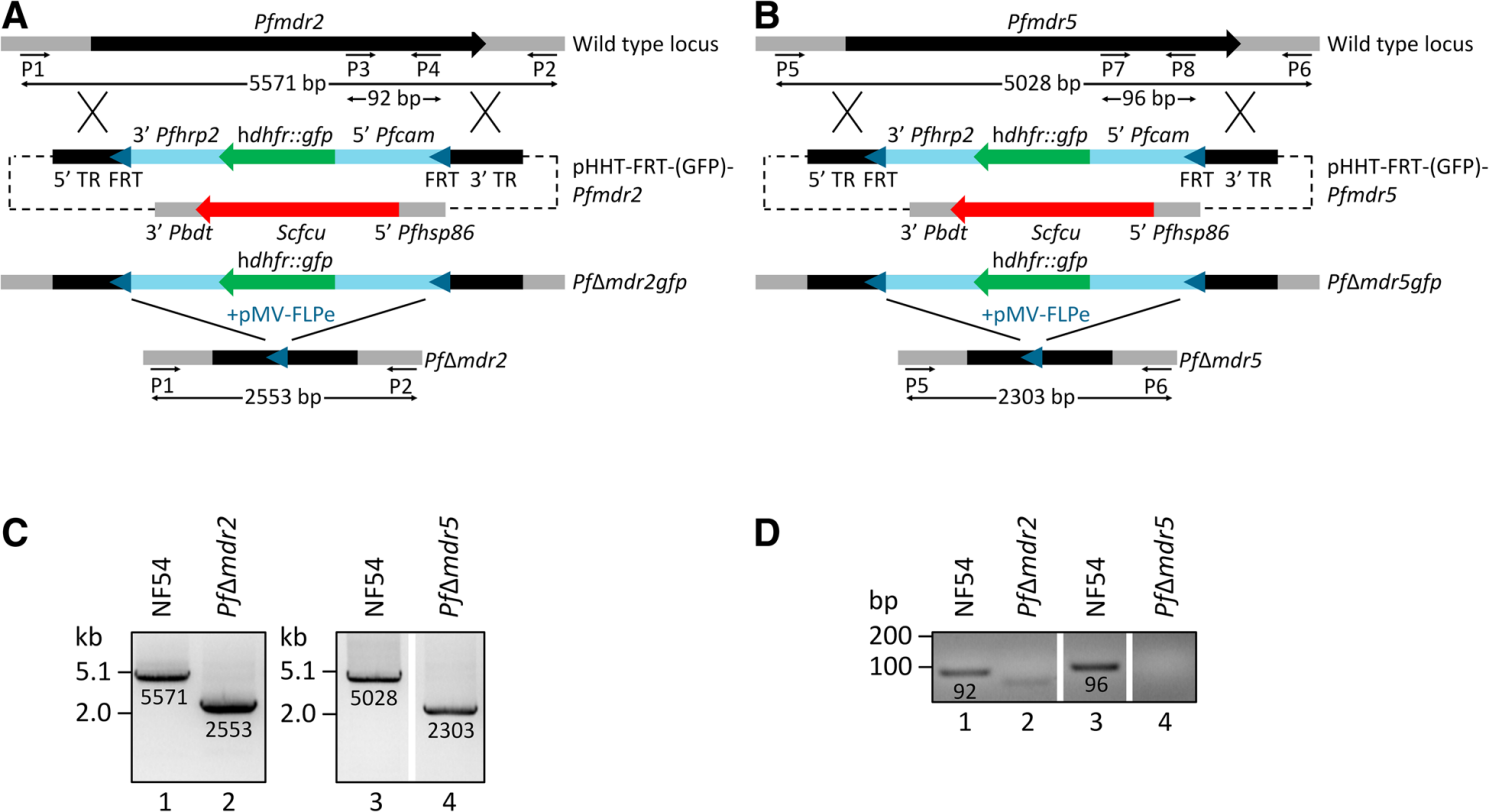
***Pfmdr2*** **and** ***Pfmdr5*** **deletion strategy and genotyping of the mutants. (A + B)** Schematic diagrams showing the *Pfmdr2* and *Pfmdr5* gene deletion strategy. Following separate transfection and positive selection on WR99210, the pHHT-FRT-(GFP)-*Pfmdr2* or pHHT-FRT-(GFP)-*Pfmdr5* construct integrated into either the 5′ or 3′ target region (TR) in the NF54 wild type locus. Upon negative selection with 5-fluorocytosine, the *mdr* gene (black arrow) was replaced by the positive selection cassette harbouring the h*dhfr::gfp* fusion gene (green arrow) flanked by FRT sites (blue triangles) (*Pf*Δ*mdr2gfp* or *Pf*Δ*mdr5gfp*). This fusion gene was subsequently removed by FLPe-mediated excision upon pMV-FLPe transfection resulting in two *mdr* deletion lines, *Pf*Δ*mdr2* and *Pf*Δ*mdr5. cam*: calmodulin; h*dhfr::gfp*: human dihydrofolate reductase fused to green fluorescent protein; *hrp*: histidine rich protein; *hsp*: heat shock protein; *mdr:* multi-drug resistance; *Scfcu*: *Saccharomyces cerevisiae* cytosine deaminase/uracil phosphoribosyl-transferase; *Pbdt*: *Plasmodium berghei dhfr* terminator; FRT: flippase recognition target; P: primer; TR: target region. Diagnostic long range **(C)** and intra-exonic **(D)** PCRs performed on genomic DNA from wild type (NF54, lane 1 or lane 3) and mutant (*Pf*Δ*mdr2*, lane 2 or *Pf*Δ*mdr5*, lane 4) lines, successfully confirmed deletion of *Pfmdr2* and *Pfmdr5* in which heterologous DNA was removed.

Possible drug resistance association of two *P. falciparum* MDR transport proteins, *Pf*MDR2 and *Pf*MDR5, were studied by a reverse genetics approach. In order to generate stable *Pfmdr2* and *Pfmdr5* gene deletion asexual stage mutants, a homologous double crossover strategy was used [28,29] with the pHHT-FRT-(GFP)-*Pfmdr2* and pHHT-FRT-(GFP)-*Pfmdr5* deletion constructs (Figure 1A and B). Heterologous DNA including the h*dhfr::gfp* selectable marker flanked by FRT sites was subsequently removed from the mutant parasites using FLP-mediated sequence excision [30]. Diagnostic long-range PCR (LR-PCR) reactions were performed to validate the lines lacking the endogenous *Pfmdr2* (*Pf*Δ*mdr2*) and *Pfmdr5* (*Pf*Δ*mdr5*) genes. LR-PCR using primers (P) P1 and P2 flanking the target regions resulted in the expected amplification products of 5,571 bp and 2,553 bp for gDNA from NF54 wild type and *Pf*Δ*mdr2* parasites, respectively (Figure 1C). Similarly, wild type and *Pf*Δ*mdr5* gDNA was amplified using P5 and P6, producing amplicons of 5,028 bp and 2,303 bp, respectively (Figure 1C). To ensure that neither of the wild type genes was still present, intra-exonic PCRs were performed using P3 and P4 plus P7 and P8 within the exons of *Pfmdr2* and *Pfmdr5*, respectively. For the *Pfmdr2* gene, PCR amplification of wild type gDNA resulted in a 92 bp product, whereas no amplification was seen in the *Pf*Δ*mdr2* line as expected (Figure 1D). Analogously, a 96 bp product was generated from wild type gDNA using the *Pfmdr5* primers, unlike *Pf*Δ*mdr5* gDNA where no product could be obtained (Figure 1D). Both generated mutants showed unaltered morphology and growth rate (data not shown). Combined, these results show that both *Pfmdr2* and *Pfmdr5* genes were successfully deleted and not required for *P. falciparum* asexual blood stage multiplication.

#### Both *Pfmdr2* and *Pfmdr5* modulate *Plasmodium falciparum* drug susceptibility

**Figure 2:**
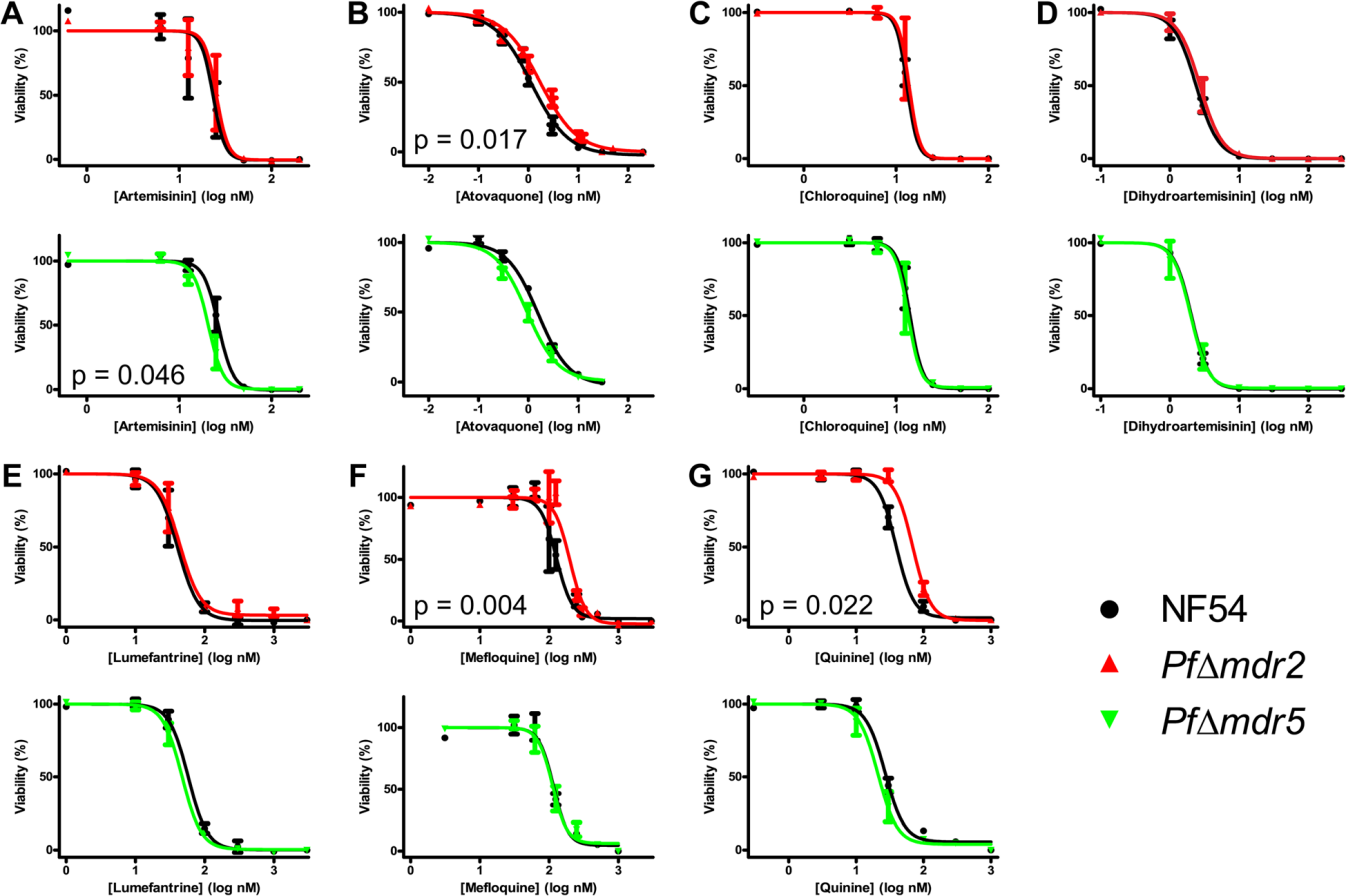
**Anti-malarial sensitivity assay of wild type and** ***Pfmdr*** **mutant parasites using seven drugs.** Dose–response inhibition curves showing sensitivity of wild type (NF54), and *P. falciparum mdr* mutant (*Pf*Δ*mdr2* and *Pf*Δ*mdr5*) parasites to ART **(A)**, ATO **(B)**, CQ **(C)**, DHA **(D)**, LUM **(E)**, MQ **(F)** and QN **(G)** were plotted with fixed Hill slopes. All assays were repeated at least three times in triplicates in order to determine significant differences by a paired *t*-test between the obtained logIC_50_-values of wild type and mutant groups.

To assess whether *Pfmdr2* and *Pfmdr5* could play a role in *P. falciparum* susceptibility to anti-malarial drugs, both *Pf*Δ*mdr2* and *Pf*Δ*mdr5* mutants were subjected to a panel containing seven drug compounds (ART, ATO, CQ, DHA, LUM, MQ, and QN) and altered sensitivity to any of the drugs was assessed compared to wild type (NF54). No difference in sensitivity to ART, CQ, DHA, and LUM was observed for the *Pf*Δ*mdr2* mutant (Figure 2A, C-E). Upon deletion of *Pfmdr2*, sensitivity to ATO (n = 7) marginally decreased, as is shown by an increase in average IC_50_ from 1.2 nM (95% CI 0.9–1.5 nM) (wild type) to 1.8 nM (95% CI 1.3–2.5 nM) (*Pf*Δ*mdr2*, p = 0.017) (Figure 2B). Furthermore, *Pf*Δ*mdr2* parasites also became less sensitive to MQ (n = 6) and QN (n = 4), both belonging to the aryl-amino alcohol class of anti-malarials [25]. For both drugs, sensitivity was reduced significantly, with an average IC_50_ of 124 nM (95% CI 86–180 nM) against MQ in wild type to 185 nM (95% CI 145–234 nM) in *Pf*Δ*mdr2* parasites (p = 0.004), and an IC_50_ of 40 nM (95% CI 27–60 nM) in wild type to 70 nM (95% CI 57–85 nM) in *Pf*Δ*mdr2* parasites for QN (p = 0.022) (Figure 2F-G). No difference in sensitivity to ATO, CQ, DHA, LUM, MQ, and QN was detected for the *Pf*Δ*mdr5* mutant (Figure 2B-G). In contrast, for ART (n = 5), the IC_50_ of *Pf*Δ*mdr5* mutant parasites decreased slightly but significantly (p = 0.046) from an average of 26 nM (95% CI 19–36 nM) in wild type to 19 nM (95% CI 14–27 nM) in *Pf*Δ*mdr5* parasites, highlighting ART as being the single compound showing an increase in sensitivity following *mdr* deletion in this study (Figure 2A).

## Discussion

Increasing *P. falciparum* resistance to anti-malarial drugs is a major threat for effective malaria treatment. For this reason, it is important to unravel proteins and mechanisms that contribute to drug resistance. While involvement of the *Pf*MDR1 transport protein in *P. falciparum* drug resistance is evident, little is known about contribution of other *P. falciparum* MDR members. Thus, using a reverse genetics approach, the aim of this study was to determine whether *Pf*MDR2 and *Pf*MDR5 could play a role in decreased drug sensitivity development using a panel of seven anti-malarials. Both *Pf*Δ*mdr2* and *Pf*Δ*mdr5* mutant lines were able to complete asexual development, highlighting that during this stage the transport proteins are dispensable for parasite viability. It is hypothesized that *Pf*Δ*mdr2* and *Pf*Δ*mdr5* become more susceptible to the tested anti-malarials if: i) the anti-malarial is a substrate for the wild type transporter; and, ii) the wild type MDR protein is located on the plasma membrane, which in the case of mutant lines would result in accumulation of the compounds within the parasite followed by its demise.

In contrast to the hypothesis above, deletion of *P. falciparum mdr2* induced some decrease in sensitivity to the anti-malarials ATO, MQ and QN. As *Pf*MDR2 has been reported to localize to the plasma membrane of the parasite [34], deleting this protein would result in accumulation of substrate anti-malarials within the parasite, leading to an increased sensitivity phenotype. However, besides plasma membrane association, *Pf*MDR2 expression on the food vacuole has also been proposed [23,35]. MQ and QN are incorporated into growing haemozoin polymers within the food vacuole [36] and therefore are toxic to the parasite, hence, deletion of *Pf*MDR2 might prevent accumulation of these drugs in the digestive vacuole, resulting in decreased sensitivity of *Pf*Δ*mdr2* to MQ and QN as shown here. In contradiction to these results, it has been reported that MQ and potentially QN prevent uptake of host cell hemoglobin by inhibiting endocytosis [37,38] and therefore it may be advantageous for *P. falciparum* to sequester these drugs in the food vacuole [39]. However, at this stage, it is unclear whether MQ and QN accumulation in the food vacuole is indeed affected by *Pfmdr2* deletion. While LUM belongs to the same drug class as MQ and QN [25], the *Pf*Δ*mdr2* mutant does not show the same trend in sensitivity to LUM as compared to the latter drugs. This might indicate that LUM is not a substrate for *Pfmdr2* or (less likely) it has a different site of action, which currently is undetermined. ATO exerts its anti-malarial activity by inhibiting the parasite’s oxygen consumption in mitochondria [40]. While mitochondrial localization of *Pf*MDR2 has not been shown, further research may link *Pf*MDR2 deletion to decreased ATO sensitivity. Moreover, it would be interesting to test several drug combinations to observe synergistic effects. These findings indicate that *Pf*MDR2 may be involved in drug sensitivity either directly or indirectly via drug substrates.

Upon deletion of *P. falciparum mdr5*, only sensitivity to ART was altered. In concordance with the hypothesis, parasites lacking *Pf*MDR5 became slightly more sensitive to ART, suggesting that this drug is a possible substrate for the *Pf*MDR5 transport protein. As *Pf*MDR5 has previously been shown to localize to the parasite’s plasma membrane [41], lacking this transport protein could lead to an accumulation of ART in the parasite, hence, resulting in increased sensitivity. On the contrary, there was no difference in sensitivity for *Pf*Δ*mdr5 versus* wild type parasites to DHA, the active metabolite of artemisinin derivatives [42], highlighting a possible subtle difference in substrate specificity.

## Conclusions

*Plasmodium falciparum* does not require *Pf*MDR2 and *Pf*MDR5 to progress through the asexual multiplication cycle. Additionally, deletion of *Pf*MDR2 resulted in a minor decreased sensitivity phenotype to ATO, MQ and QN. Therefore, *P. falciparum* might increase its resistance to these anti-malarials by modulating *Pf*MDR2 in order to prevent these drugs from reaching their proposed target sites. Moreover, the *Pf*MDR5 deletion mutant became slightly more sensitive upon exposure to ART, suggesting that over-expression or enhanced activity of this protein might contribute to ART resistance.

## Additional file

Additional file 1: Table S1. Diagnostic long-range primers, intra-exonic primers and targeting construct primers.

## References

[1] Higgins CF (1992). ABC transporters: from microorganisms to man. Annu Rev Cell Biol.

[2] Rees DC, Johnson E, Lewinson O (2009). ABC transporters: the power to change. Nat Rev Mol Cell Biol.

[3] Walker JE, Saraste M, Runswick MJ, Gay NJ (1982). Distantly related sequences in the alpha- and beta-subunits of ATP synthase, myosin, kinases and other ATP-requiring enzymes and a common nucleotide binding fold. EMBO J.

[4] Hyde SC, Emsley P, Hartshorn MJ, Mimmack MM, Gileadi U, Pearce SR (1990). Structural model of ATP-binding proteins associated with cystic fibrosis, multidrug resistance and bacterial transport. Nature.

[5] Gottesman MM, Hrycyna CA, Schoenlein PV, Germann UA, Pastan I (1995). Genetic analysis of the multidrug transporter. Annu Rev Genet.

[6] Gottesman MM, Pastan I, Ambudkar SV (1996). P-glycoprotein and multidrug resistance. Curr Opin Genet Dev.

[7] Ling V, Gerlach J, Kartner N (1984). Multidrug resistance. Breast Cancer Res Treat.

[8] Koenderink JB, Kavishe RA, Rijpma SR, Russel FG (2010). The ABCs of multidrug resistance in malaria. Trends Parasitol.

[9] WHO (2014). Malaria Fact sheet N°94.

[10] Dondorp AM, Nosten F, Yi P, Das D, Phyo AP, Tarning J (2009). Artemisinin resistance in *Plasmodium falciparum* malaria. N Engl J Med.

[11] Amaratunga C, Sreng S, Suon S, Phelps ES, Stepniewska K, Lim P (2012). Artemisinin-resistant *Plasmodium falciparum* in Pursat province, western Cambodia: a parasite clearance rate study. Lancet Infect Dis.

[12] Ariey F, Witkowski B, Amaratunga C, Beghain J, Langlois AC, Khim N (2014). A molecular marker of artemisinin-resistant *Plasmodium falciparum* malaria. Nature.

[13] Foote SJ, Thompson JK, Cowman AF, Kemp DJ (1989). Amplification of the multidrug resistance gene in some chloroquine-resistant isolates of *P. falciparum*. Cell.

[14] Peel SA, Bright P, Yount B, Handy J, Baric RS (1994). A strong association between mefloquine and halofantrine resistance and amplification, overexpression, and mutation in the P-glycoprotein gene homolog (pfmdr) of *Plasmodium falciparum* in vitro. Am J Trop Med Hyg.

[15] Raj DK, Mu J, Jiang H, Kabat J, Singh S, Sullivan M (2009). Disruption of a *Plasmodium falciparum* multidrug resistance-associated protein (PfMRP) alters its fitness and transport of antimalarial drugs and glutathione. J Biol Chem.

[16] Veiga MI, Ferreira PE, Jornhagen L, Malmberg M, Kone A, Schmidt BA (2011). Novel polymorphisms in *Plasmodium falciparum* ABC transporter genes are associated with major ACT antimalarial drug resistance. PLoS One.

[17] Hao M, Jia D, Li Q, He Y, Yuan L, Xu S (2013). In vitro sensitivities of *Plasmodium falciparum* isolates from the China-Myanmar border to piperaquine and association with polymorphisms in candidate genes. Antimicrob Agents Chemother.

[18] Phompradit P, Muhamad P, Wisedpanichkij R, Chaijaroenkul W, Na-Bangchang K (2014). Four years’ monitoring of in vitro sensitivity and candidate molecular markers of resistance of *Plasmodium falciparum* to artesunate-mefloquine combination in the Thai-Myanmar border. Malar J.

[19] Mu J, Ferdig MT, Feng X, Joy DA, Duan J, Furuya T (2003). Multiple transporters associated with malaria parasite responses to chloroquine and quinine. Mol Microbiol.

[20] Anderson TJ, Nair S, Qin H, Singlam S, Brockman A, Paiphun L (2005). Are transporter genes other than the chloroquine resistance locus (pfcrt) and multidrug resistance gene (pfmdr) associated with antimalarial drug resistance?. Antimicrob Agents Chemother.

[21] Okombo J, Abdi AI, Kiara SM, Mwai L, Pole L, Sutherland CJ (2013). Repeat polymorphisms in the low-complexity regions of *Plasmodium falciparum* ABC transporters and associations with in vitro antimalarial responses. Antimicrob Agents Chemother.

[22] Wang Z, Parker D, Meng H, Wu L, Li J, Zhao Z (2012). In vitro sensitivity of *Plasmodium falciparum* from China-Myanmar border area to major ACT drugs and polymorphisms in potential target genes. PLoS One.

[23] Rosenberg E, Litus I, Schwarzfuchs N, Sinay R, Schlesinger P, Golenser J (2006). pfmdr2 confers heavy metal resistance to *Plasmodium falciparum*. J Biol Chem.

[24] Briolant S, Bogreau H, Gil M, Bouchiba H, Baret E, Amalvict R (2012). The F423Y mutation in the pfmdr2 gene and mutations N51I, C59R, and S108N in the pfdhfr gene are independently associated with pyrimethamine resistance in *Plasmodium falciparum* isolates. Antimicrob Agents Chemother.

[25] Rosenthal PJ (2013). The interplay between drug resistance and fitness in malaria parasites. Mol Microbiol.

[26] Ifediba T, Vanderberg JP (1981). Complete in vitro maturation of *Plasmodium falciparum* gametocytes. Nature.

[27] Ponnudurai T, Lensen AH, Leeuwenberg AD, Meuwissen JH (1982). Cultivation of fertile *Plasmodium falciparum* gametocytes in semi-automated systems. 1. Static cultures. Trans R Soc Trop Med Hyg.

[28] Duraisingh MT, Triglia T, Cowman AF (2002). Negative selection of *Plasmodium falciparum* reveals targeted gene deletion by double crossover recombination. Int J Parasitol.

[29] Maier AG, Braks JA, Waters AP, Cowman AF (2006). Negative selection using yeast cytosine deaminase/uracil phosphoribosyl transferase in *Plasmodium falciparum* for targeted gene deletion by double crossover recombination. Mol Biochem Parasitol.

[30] van Schaijk BC, Vos MW, Janse CJ, Sauerwein RW, Khan SM (2010). Removal of heterologous sequences from *Plasmodium falciparum* mutants using FLPe-recombinase. PLoS One.

[31] Su XZ, Wu Y, Sifri CD, Wellems TE (1996). Reduced extension temperatures required for PCR amplification of extremely A + T-rich DNA. Nucleic Acids Res.

[32] Gamo FJ, Sanz LM, Vidal J, de Cozar C, Alvarez E, Lavandera JL (2010). Thousands of chemical starting points for antimalarial lead identification. Nature.

[33] Jensen JB, Trager W (1977). *Plasmodium falciparum* in culture: use of outdated erthrocytes and description of the candle jar method. J Parasitol.

[34] Rubio JP, Cowman AF (1994). *Plasmodium falciparum*: the pfmdr2 protein is not overexpressed in chloroquine-resistant isolates of the malaria parasite. Exp Parasitol.

[35] Zalis MG, Wilson CM, Zhang Y, Wirth DF (1993). Characterization of the pfmdr2 gene for *Plasmodium falciparum*. Mol Biochem Parasitol.

[36] Mungthin M, Bray PG, Ridley RG, Ward SA (1998). Central role of hemoglobin degradation in mechanisms of action of 4-aminoquinolines, quinoline methanols, and phenanthrene methanols. Antimicrob Agents Chemother.

[37] Hoppe HC, van Schalkwyk DA, Wiehart UI, Meredith SA, Egan J, Weber BW (2004). Antimalarial quinolines and artemisinin inhibit endocytosis in *Plasmodium falciparum*. Antimicrob Agents Chemother.

[38] Famin O, Ginsburg H (2002). Differential effects of 4-aminoquinoline-containing antimalarial drugs on hemoglobin digestion in *Plasmodium falciparum*-infected erythrocytes. Biochem Pharmacol.

[39] Rohrbach P, Sanchez CP, Hayton K, Friedrich O, Patel J, Sidhu AB (2006). Genetic linkage of pfmdr1 with food vacuolar solute import in *Plasmodium falciparum*. EMBO J.

[40] Murphy AD, Lang-Unnasch N (1999). Alternative oxidase inhibitors potentiate the activity of atovaquone against *Plasmodium falciparum*. Antimicrob Agents Chemother.

[41] Kavishe RA, van den Heuvel JM, van de Vegte-Bolmer M, Luty AJ, Russel FG, Koenderink JB (2009). Localization of the ATP-binding cassette (ABC) transport proteins PfMRP1, PfMRP2, and PfMDR5 at the *Plasmodium falciparum* plasma membrane. Malar J.

[42] Navaratnam V, Mansor SM, Sit NW, Grace J, Li Q, Olliaro P (2000). Pharmacokinetics of artemisinin-type compounds. Clin Pharmacokinet.

